# Shark Tooth Weapons from the 19^th^ Century Reflect Shifting Baselines in Central Pacific Predator Assemblies

**DOI:** 10.1371/journal.pone.0059855

**Published:** 2013-04-03

**Authors:** Joshua Drew, Christopher Philipp, Mark W. Westneat

**Affiliations:** 1 Division of Fishes and Biodiversity Synthesis Center, Field Museum of Natural History, Chicago, Illinois, United States of America; 2 Department of Ecology, Evolution and Environmental Biology, Columbia University, New York, New York, United States of America; 3 Department of Anthropology, Field Museum of Natural History, Chicago, Illinois, United States of America; New York State Museum, United States of America

## Abstract

The reefs surrounding the Gilbert Islands (Republic of Kiribati, Central Pacific), like many throughout the world, have undergone a period of rapid and intensive environmental perturbation over the past 100 years. A byproduct of this perturbation has been a reduction of the number of shark species present in their waters, even though sharks play an important in the economy and culture of the Gilbertese. Here we examine how shark communities changed over time periods that predate the written record in order to understand the magnitude of ecosystem changes in the Central Pacific. Using a novel data source, the shark tooth weapons of the Gilbertese Islanders housed in natural history museums, we show that two species of shark, the Spot-tail (*Carcharhinus sorrah*) and the Dusky (*C. obscurus*), were present in the islands during the last half of the 19^th^ century but not reported in any historical literature or contemporary ichthyological surveys of the region. Given the importance of these species to the ecology of the Gilbert Island reefs and to the culture of the Gilbertese people, documenting these shifts in baseline fauna represents an important step toward restoring the vivid splendor of both ecological and cultural diversity.

## Introduction

Understanding historical ecological conditions is an important first step in evaluating conservation measures [1]. However, in systems where empirically derived ecological data are lacking, we must rely on non-traditional measures of community composition [2]. Examples of non-traditional data sources include fisher interviews [3], [4], historical photographs [5], fisheries’ catch records [6], and the archaeological record [7], [8]. In some cases, the material culture of indigenous peoples may represent an underutilized source of data. These data, when housed in natural history museum collections, represent spatially and temporally fixed collection points [9]. By examining the materials used in constructing these items, we can gain access to the flora and fauna present during the time of their construction, and when these materials are assigned to a particular species, they can indicate which species were present in the past. We are able to track the changes in community composition through time by comparing these records with modern faunal lists.

Sharks are an important component in Central Pacific ecosystems [10] where they influence the trophic structure of reefs [11] and the behavioral ecology of the species living on those reefs [12], [13]. Despite their ecological importance, sharks globally are facing several conservation threats, including directed [14] and non-directed fisheries pressure [15]. A major source of mortality comes from a directed fishery for shark fins, often for sale in Asian markets as the key ingredient of shark fin soup [16]. Because of these threats, shark populations are declining globally, and they have dropped by as much as 99% in areas where there is active fishing pressure [17] and 90% within no-take marine protected areas, which are subject to poaching [18].

**Figure 1:**
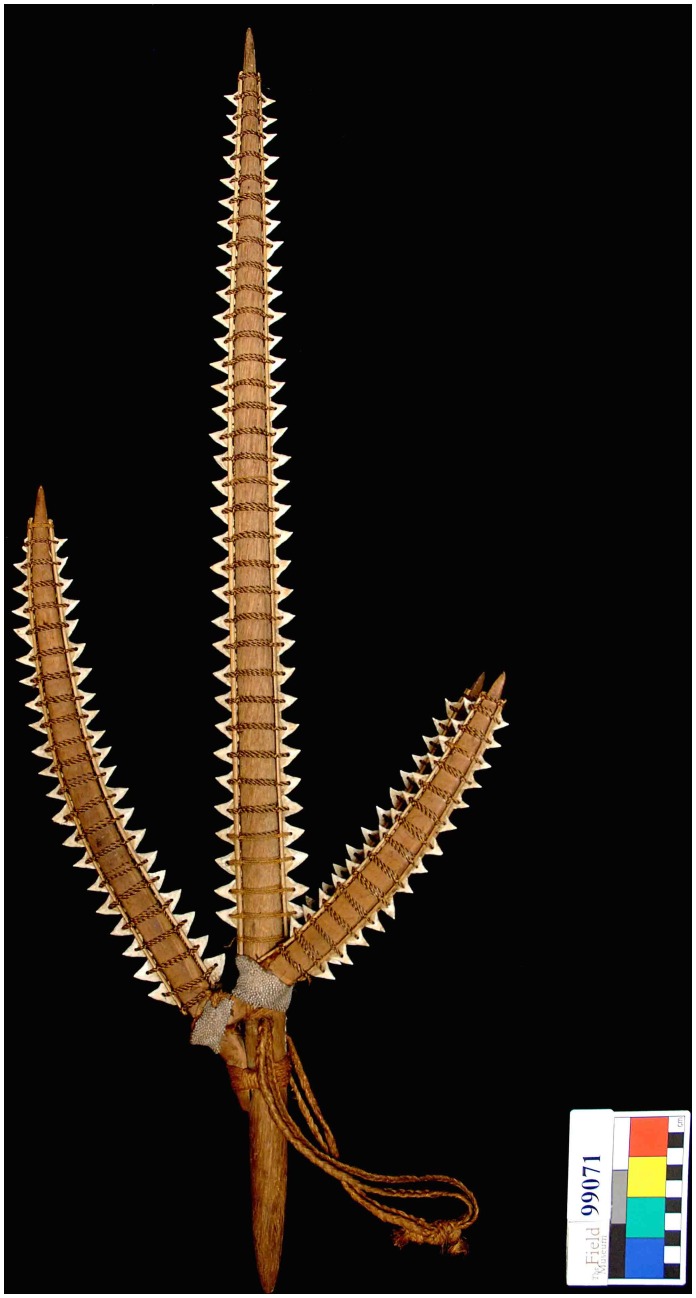
An example of a Gilbertese shark tooth weapon (FMNH 99071).

**Figure 2:**
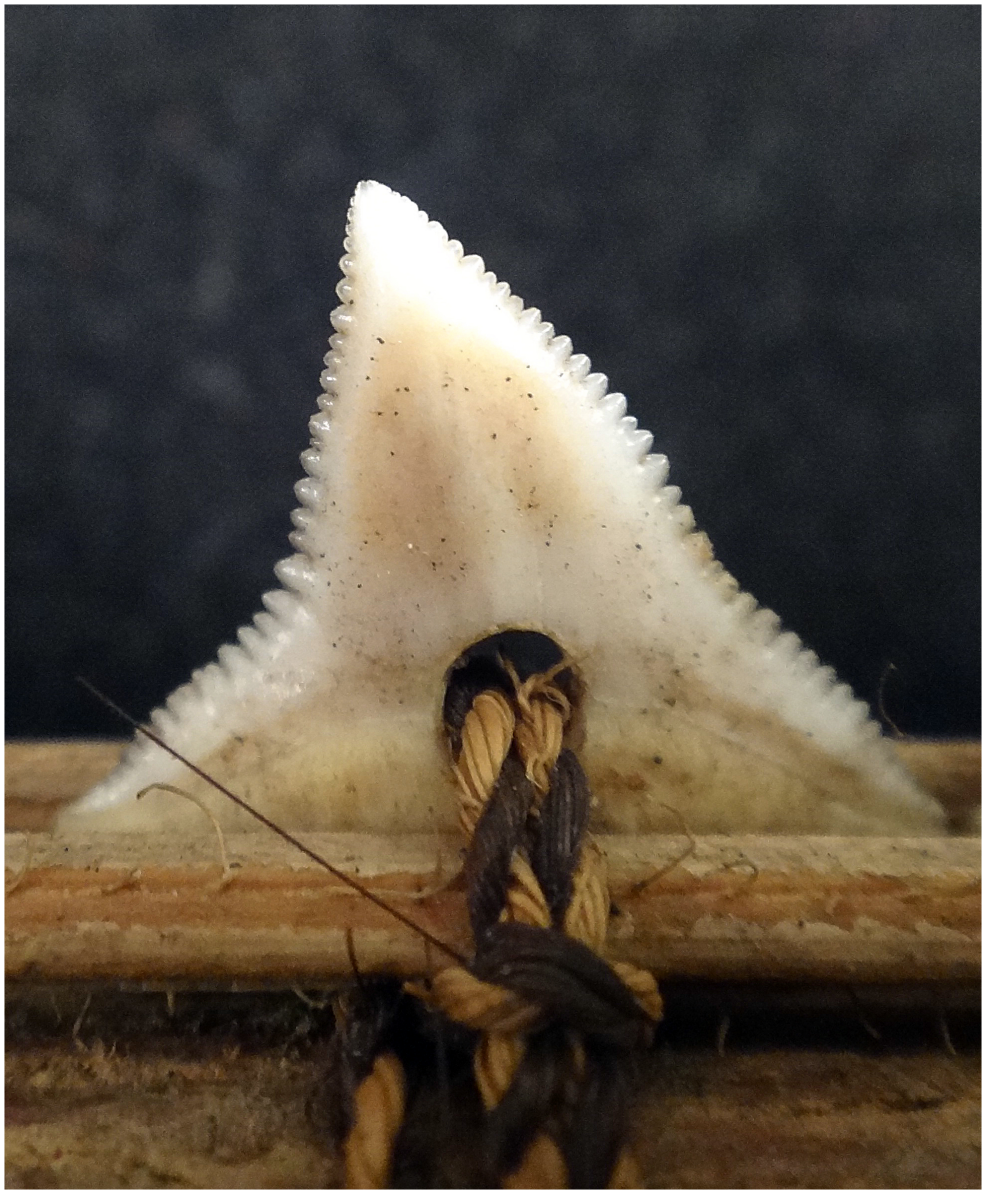
Close up of FMNH 99071 showing how the teeth of *Carcharhinus obscurus* were attached using braded cord.

Sharks are culturally significant in the worldview of indigenous Gilbertese people who inhabit the Gilbert Islands. Sharks feature prominently in their settlement mythology [19] and in historical ceremonies to initiate boys into adulthood [20]. Historically, the Gilbertese Islanders had an extensive knowledge of shark biology and evolved a complex ritual system surrounding shark fishing [21] and produced a diverse array of fishing gear [21], [22]. They also created a suite of characteristic shark tooth weapons [23]. The literature is silent on when these weapons were first made, but they were in production at the time of the first Western contact [24]. These weapons were present across many different groups in the Gilbert Society and included a wide range of types such as daggers, swords, and spears that were used in highly ritualized, and often fatal, conflicts [23], [25]. To construct these weapons, small transverse holes were drilled into individual shark teeth, and the teeth were affixed to pieces of wood with braided coconut fibers that were often combined or intertwined with strands of human hair (Fig. 1, 2); these pieces of wood were then attached to the main shaft of the weapon. These weapons were the focus of intensive collecting by natural history museums throughout the mid to late 19^th^ century.

Because shark teeth are largely diagnostic to species, these anthropological holdings allow us to identify some species of sharks present in the Gilbert Islands waters during the period of these weapons’ manufacture (1840–1898). When combined with historical records, these identifications allow us to reconstruct the shark community of the Gilbert Islands. In doing so, we are able to identify how the baseline of an apex predatory community has shifted over time.

## Materials and Methods

### Weapon Analysis

We took data from Gilbertese weapons accessioned in the Anthropology holdings of the Field Museum of Natural History (FMNH). There are 124 shark tooth weapons in the collection. The weapons sampled can be broken down into the following categories: 1 “club,” 24 “daggers,” 2 “knives,” 2 “lances,” 15 “spears,” 21 “swords,” and 59 unidentified. The collection also contained 16 individual or multiple shark teeth lots and 1 tattooing instrument equaling 17 non-weapons. In all, we collected data from 122 objects, including individual teeth and weapons.

For each sample (Table S1), we photographed the weapon as a whole and then took high-resolution photographs of individual shark teeth. We also took photographs of 8–12 teeth from each weapon and at least one photo of each different species present in each weapon. Gross tooth morphology, which includes tooth shape, serration pattern, and pattern of supporting elements on the tooth, was then compared to illustrations in the FAO guides to sharks, the most universally accepted references for sharks in the orders Carcharhiniformes [26] and Lamniformes [27]. In addition, we independently validated species’ dentition using the ichthyology holdings of the FMNH and the American Museum of Natural History (AMNH – see Table S2).

### Literature Analysis

To complement the temporally limited data set provided by the weapons (1848–1898), we also performed a literature search to generate a list of shark species reported within the Gilbert Islands waters (Table 1). We divided the literature into historical (prior to 1985) and contemporary (1985 to present). These reports are spatially variable with several different islands within the group sampled; thus, the results represent an archipelagic aggregate of biodiversity. In addition, they were generated with a variety of different sampling and methodologies and are therefore not internally comparable. Invalid species names were evaluated using the Eschmeyer Catalogue of fishes housed at the California Academy of Science (http://researcharchive.calacademy.org/research/Ichthyology/catalog/fishcatmain.asp). Current shark species composition was compiled using FishBase [28]. As FishBase only aggregates data by country, the sharks listed are for all of Kiribati, not just the Gilbert Islands, and therefore should be considered a maximum estimate of species diversity. To help reduce this bias, fishes that were explicitly listed in FishBase as questionable for the country as a whole, or as specifically being found in one of the other archipelagos within Kiribati (Phoenix or Line islands), were excluded from our species list. We acknowledge that this data source is of variable quality [29], and to help address these issues of data quality, we also incorporated data from published ichthyologic research within the region [12].

**Table 1:** A list of sharks recorded in the Gilbert Islands based on data from teeth, historical (pre 1986) and contemporary (post 1985) literature.

Species	Common Name	Local Name	Citation	Weapons	Historical Lit	Contemperary lit
*Alopias vulpinus*	Common thrasher shark		Bingham (1908) inLoumala (1984)		x	x
*Carcharhinus albimarginatus*	Silvertip Shark	Te bakoa	FishBase	x		x
*Carcharhinus amblyrhynchos*	Grey Reef Shark	Te bakoanimarawa	FishBase			x
*Carcharhinus falciformis*	Silky Shark		FishBase	x	x	x
*Carcharhinus longimaus*	Oceanic White Tip Shark		FishBase	x		x
*Carcharhinus melanopterus*	Black Tip Reef Shark	Te baiburebure	FishBase		x	x
*Carcharhinus obscurus*	Dusky shark			x		
*Carcharhinus sorrah*	Spotfin shark			x		
*Galeocerdo cuvier*	Tiger Shark	Te tababa	FishBase	x	x	x
*Isistius brasiliensis*	Cookiecutter shark		FishBase			x
*Isurus oxyrihchus*	Shortfin Mako		Loumala (1984)		x	x
*Isurus paucus*	Longfin Mako		Loumala (1984)		x	x
*Nebrius ferrugineus*	Tawnny Nurse Shark	Bákoa	Randall (1955)		x	x
*Negaprion acutidens*	Sicklefin lemon shark	Te unun	Randall (1955)		x	x
*Prionace glauca*	Blue Shark		Lampert (1968)	x	x	x
*Rhincodon typus*	Whale Shark		FishBase			x
Sphyrnidae sp.	Hammerhead sharks		Whitley (1938)	x	x	
*Triaenodon obesus*	White Tip Reef Shark	Te bakoa	FishBase		x	x

### Collections Analysis

We searched the collection databases of several major natural history museums (Academy of Natural Sciences Philadelphia; American Museum of Natural History; Australian Museum; California Academy of Sciences; LA County Museum; Museum of Comparative Zoology; Natural History Museum, London; U.S. National Museum of Natural History; and University of Washington Fish Collection) for fishes collected in the Gilbert Islands. We collected the year, approximate time spent collecting and the location of the collections made in these expeditions (Table 2) as well as recording any sharks that were from the Gilbert Islands that are currently (or historically) in holdings (Table 3).

**Table 2:** A list of collections made from the Gilbert Islands.

Institution(s)	Year	Days spent collecting	Localties
MCZ ANS	1860–1883	Sporadic collections from A. Garrett	Kingsmill
NHM	1873	Unspecified	Tarawa
ANM, NMNH	1951	62	Tarawa
Australian Museum	1955	Sporadic collections from R. Catala	Arorae, Tarawa
UWFC	1956	10	Tarawa
Australian Museum	1962–1963	Unspecified	Apoiang Atoll
NHM	1969	Unspecified	Tarawa
Multiple Institutions	1973	10	Abaiang Atoll

Data presented are from major collections (>25 specimens) and were gathered from Fishnet2.net and respective on-line catalogues. Abbreviations are as follows: ANM (Australian National Museum), ANS (Academy of Natural Sciences), NHM (Natural History Museum of London), NMNH (National Museum of Natural History), UWFC (University of Washington Fish Collection).

**Table 3:** Sharks from the Gilbert Islands in Museum Holdings.

Species	Year	Collections number	Collection locality
*C. falciformes*	1951	USNM 167438	Onotoa Atoll
*Carcharias sp.*	1860	MCZ S-113	Kingmill Islands
*Scoliodon melanopterus* *	1860	MCZ S-112	Kingmill Islands
*Triaenodon apicalis*	1973	ANM I.18056-001	Abaiang Atoll

* specimen has been lost. Most likely synonomized with C. melanopterus.

## Results

### Weapons Analysis

Although species-specific tooth morphology could not be assessed on every weapon because of tooth size or damage from conflict, we were able to identify teeth from the following eight species: *Carcharhinus albimarginatus, C. falciformis, C. longimaus, C. obscurus, C. sorrah, Galeocerdo cuvier*, *Prionace glauca,* and *Sphyrna zygaena*. Two of these, *C. obscurus* and *C. sorrah*, were not recorded in any other data source as having occurred in the Gilbertese waters. Of the sampled weapons, *C. albimarginatus* was the most frequently encountered shark, appearing in 34 weapons; *C. obscurus* was found in 29 items; and *C. sorrah* was present in 6 items.

### Literature Analysis

Contemporary data report 15 species of shark (Table 1). Historical literature, published between 1908 and 1984, lists 11 species. The data from the historical literature is not a nested subset of the contemporary data. Three species of sharks present in the contemporary literature data set are not present in the historical literature data set (*C. albimarginatus, C. amblyrhinchos,* and *C. longimaus*), and one group present in the historical literature, Hammerhead sharks (family *Sphyrnidae*), was not listed in the contemporary literature.

### Collections Analysis

There have been eight major collections from the Gilbert Islands, six for museum expeditions and two made by researchers from the area (Table 2). Only one of these reported collecting a shark (a *C. falciformis* caught by a Gilbertese fisherman in 1951 and acquired by Jack Randall for the USNM expedition). The search of collections databases of these museums found only four sharks that originated in the Gilbert Islands (Table 3). Interestingly, one of these (MCZ S-113) was collected in 1860 and represents the only record of the genus *Carcharias* for the Gilbert Islands. However, because there is only one record of this genus, its inclusion in the list of native shark fauna should be considered tentative until supporting evidence is considered. Similarly MCZ S-112, also collected in 1860, is now lost and its identification remains tentative.

## Discussion

Our results show that two species of sharks that were once present in the Gilbert Islands are no longer recognized as being part of the extant reef community. Moreover, as these species are common throughout their current range and are commercially important [26], it is unlikely (but not impossible) that these species have been overlooked in contemporary ichthyological surveys. An alterative possibility is that these shark species have been extirpated from the region. Regardless of which of these two hypotheses is supported, the identification of these species as being present in the Gilbert Islands waters at least 130 years ago highlights how the baseline of apex predatory community has shifted to no longer include these two species. Because of an increase in sampling efforts and improvements in technology, the number of recorded shark species in the Gilberts has increased over time. Our findings from the weapons show that, despite this increased sampling effort and ability, two species that were once present are now absent from these waters.

Unfortunately, the data do not allow us to make estimates of relative shark diversity in 19^th^ century Gilbert reefs because we do not know if fishers were preferentially targeting species or sampling randomly among those available. Thus, it is impossible to say, for example, if the rarity of *C. sorrah* teeth in the weapons collection represents a relative scarcity of individuals in the Gilbert Islands waters or the fishers’ preference for different species’ teeth. Given the lack of small teeth present in weapons, and the need for these teeth to stand up to the rigors of combat, it is likely that there was some active decision making in the selection of size and species of sharks to target.

While doing this research, we made the assumption that the shark teeth present in the weapons were derived locally. Although it is possible that there was trade between the Gilbertese people and those living in areas where *C. obscurus* and *C. sorrah* are currently found, it is likely that these fish were collected by the Gilbertese people. Two lines of evidence support this claim. First, there are no records among the historical, archaeological, or linguistic literature of exchange among the Gilbert Islands and people in the Solomon Islands (the nearest location for *C. sorrah*) or Fiji (for *C. obscurus*). Second, the Gilbertese had a well-developed shark fishery using a variety of techniques and exploiting a variety of habitats [21], reducing the need to import a locally obtainable resource. These techniques included deep water hook and line fisheries, including some hooks being set at over 300 fathoms (549 m), which is well within the depth ranges of all these species [21], [22]. This lack of evidence of trade from areas where the sharks are currently found, combined with a well-documented material technology and methodology for capturing sharks *in situ* suggest that the simplest explanation for the presence of these teeth in Gilbertese weaponry is that they were harvested locally.

Sharks possess a number of biological characteristics (slow growth, late age of maturity, low fecundity) that predispose them to extirpation by fishing [30]. There are reports of commercial shark fishing for the fin industry in the Gilbert Islands as far back as 1910 [21], and by the 1950s, over 3,000 kg of shark fins were being taken from the Gilberts annually [31]. Shark populations can be driven to extinction quickly with even moderate levels of fishing pressure. Luiz and Edwards [13] found that populations of the closely related *C. galapagensis* and *C. falciformis* in a remote Atlantic archipelago were extirpated in less than 40 years after the start of a commercial fishery.

Our findings demonstrate that our understanding of the species dwelling within this marine ecosystem has shifted over the past 130 years and that two former components of that system are no longer present. The combined data from weapons, literature, and museum collections show how increases in sampling effort and technology have allowed us to better explore the oceans and to characterize its denizens. However, nested within this story is a cautionary tale of how species’ presence is mutable and how what we see today is not necessary indicative of the past.

This alteration of predator communities can affect both biological and cultural diversity because sharks play an important role in structuring marine ecosystems. The historical loss of two species from the Gilbert Islands may impact both the functioning and the resilience of the contemporary ecosystem. Additionally, sharks once played an important part in the culture of the Gilbertese people, and the loss of these species may have obvious or subtle impacts on the social structuring, material culture, and worldview of the Gilbertese people [19]–[21], [23], [32]–[34].

When baseline perceptions are shifted to a more degraded state, they hamper conservation actions by providing goals that may be less ambitious or less capable of true conservation and restoration [35]. In order for conservation measures to recapture the vivid splendor of past coral reefs, it is critical to describe what a healthy reef community looked like in the past. By incorporating historical information, we are better able to evaluate how successful conservation measures are and set more biologically realistic conservation targets. However, one of the more insidious aspects of the shifting baseline syndrome [2] is a cultural amnesia, where people forget how vibrant reefs really were. Here we demonstrate how using non-traditional forms of data can help reconstruct the apex predator community, identify shadow biodiversity–biodiversity that has been lost before formal scientific assessment–and, we hope, prevent an otherwise inexorable decline in both the biological and cultural diversity of Central Pacific islands.

Our results also underscore the importance of maintaining collections and the value of providing these data to a wide variety of audiences [9]. The value of natural history collections lies in their spatially and temporally explicit nature and in their ability to serve as baselines for future studies [36], and this case illustrates the value of archiving information for future use. As museums move towards digitizing their collections, we anticipate the importance of collections will increase, and we argue for funds to be allocated for their continued development and maintenance.

## Supporting Information

Table S1 A list of Shark Tooth Weapons in the FMNH collections used in this study.
(XLSX)

Table S2 A list of shark jaws in the FMNH and AMNH collections used to evaluate the weapons from Table S1.
(XLSX)
